# Resveratrol-nitric oxide donor hybrid effect on priapism in sickle cell and nitric oxide-deficient mouse

**DOI:** 10.1371/journal.pone.0269310

**Published:** 2022-06-02

**Authors:** Andressa Kely Pinheiro, Dalila Andrade Pereira, Jean Leandro dos Santos, Fabiano Beraldi Calmasini, Eduardo Costa Alexandre, Leonardo Oliveira Reis, Arthur L. Burnett, Fernando Ferreira Costa, Fábio Henrique Silva

**Affiliations:** 1 Laboratory of Multidisciplinary Research, São Francisco University Medical School, Bragança Paulista, SP, Brazil; 2 State University of São Paulo (UNESP), School of Pharmaceutical Science, Laboratory of Drug Discovery, Araraquara, SP, Brazil; 3 Department of Structural and Functional Biology, University of Campinas, Campinas, SP, Brazil; 4 Department of Pharmacology, Faculty of Medical Sciences, University of Campinas, Campinas, SP, Brazil; 5 UroScience, Pontifical Catholic University of Campinas, Campinas, SP, Brazil; 6 The James Buchanan Brady Urological Institute and Department of Urology, The Johns Hopkins School of Medicine, Baltimore, MD, United States of America; 7 Hematology and Hemotherapy Center, University of Campinas, Campinas, SP, Brazil; PLOS ONE, UNITED KINGDOM

## Abstract

**Background:**

Children and adult with sickle cell disease (SCD) display priapism associated with low nitric oxide (NO) bioavailability and oxidative stress in penis.

**Aim:**

This study aimed to evaluate the effects of hybrid compound RVT-FxMe, derived from resveratrol bearing a NO-donor subunit, on two murine model that display priapism phenotype, SCD transgenic mice and endothelial NO synthase gene-deficient (eNOS^-/-^) mice.

**Methods:**

Wild-type, SCD, and eNOS^-/-^ mice were treated with RVT-FxMe (25 mg/kg/d, 2 weeks).

**Outcomes:**

Hematological parameters, concentration-response curves to acetylcholine (ACh) and sodium nitroprusside (SNP), as well as to electrical field stimulation (EFS), were obtained in mice corpus cavernosum strips.

**Results:**

Corpus cavernosum relaxations to SNP and EFS were increased in eNOS^-/-^ group, which were normalized by RVT-FxMe treatment. SCD mice exhibited an excessive CC relaxant response induced by ACh, EFS and SNP RVT-FxMe treatment did not change the increased relaxant responses to ACh, EFS and SNP in corpus cavernosum from SCD group.

**Clinical translation:**

Excess of plasma hemoglobin in SCD may interfere in pharmacological activity of NO donors compounds.

**Strength/Limitations:**

While mechanistic data with promising potential is showed, the current study is not without limitations. RVT-FxMe effects in the mid- and long-term warrant complementary studies.

**Conclusion:**

Treatment with RVT-FxMe reversed the enhanced NO-cGMP-mediated CC relaxations in eNOS^-/-^ mice, but not in SCD mice; it is likely that excess of plasma hemoglobin in SCD mice act to inactivate NO before it reaches soluble guanylyl cyclase, avoiding restoration of NO bioavailability in penis.

## Introduction

Penile erection is basically initiated by corpus cavernosum (CC) smooth muscle relaxation [1]. Nitric oxide (NO)-cyclic guanosine monophosphate (cGMP) signaling pathway is the most important inducer of erectile tissue relaxation [2]. NO is produced by endothelial nitric oxide synthase (eNOS) and neuronal nitric oxide synthase (nNOS) from endothelial cells and nitrergic fibers in penis, respectively. NO stimulates soluble guanylyl cyclase (sGC) that converts GTP to cGMP, resulting in stimulation of cGMP-dependent protein (PKG), which promotes CC smooth muscle cells relaxation [1]. In smooth muscle, cGMP is quickly converted to 5’GMP by phosphodiesterase type5 (PDE5), thus finishing erectile response [2].

Sickle cell disease (SCD) is an autosomal recessive disorder that occurs by a mutation in the β-globin gene [3]. This alteration results in the production of an abnormal hemoglobin, referred to as hemoglobin S (HbS). HbS in the deoxygenated state forms polymers within red blood cells, making them rigid and resulting in hemolysis, severe hemolytic anemia, vaso-occlusive crisis, leg ulcers, pulmonary hypertension, stroke and priapism [3]. Transgenic mouse models of SCD are used to study the pathophysiology of this disease and new pharmacological treatments. Townes and Berkeley SCD mice model are widely studied in basic sciences because express them 100% HbS in their circulating erythrocytes and exhibit many features found in human SCD such as vaso-occlusion, organ damage and priapism phenotype [4–7].

Priapism is a clinical issue that frequently occurs in children and adult with SCD [8]. Priapism is defined as a penile erection that persists beyond, or is unrelated, to sexual interest or stimulation that may progress to erectile dysfunction [6]. Experimental studies have reported that dysregulation of NO-cGMP-PDE5 signaling pathway in penises of men [9] and animals, both with SCD, is associated with priapism [6, 7, 10–12]. The most important alteration found in those conditions was a downregulation of both eNOS and PDE5 [6, 7, 10–12]. In SCD and eNOS gene-deficient (eNOS^-/-^) mice CC, low NO-cGMP bioavailability is associated with PDE5 downregulation [6, 10–12]. In fact, experimental studies have shown that *in vitro* NO signaling stimulation induced by ACh and electrical-field stimulation results in increased cavernosal relaxations in SCD and eNOS^-/-^ mice due PDE5 downregulation [6, 7, 11, 13]. The *in vitro* addition of the vasoactive agent adenosine also produces exacerbated CC relaxation in SCD mice [14].

The SCD increases reactive-oxygen species production and reduces antioxidant capacity of the cells, leading to tissue injury [15]. Oxidative and nitrosative stresses are increased in penises from men and mice with SCD [6, 9, 11]. The NADPH oxidase enzyme is the most important source of superoxide anion in vascular cells. Molecular studies have shown increased NADPH oxidase subunit gp91^phox^ protein expression in CC from SCD mice and men [6, 9, 16]. Excess of superoxide anion reacts with NO to form peroxynitrite, an even more toxic specie [17].

Resveratrol (trans-3,5,4′-trihydroxystilbene) is a natural phytoalexin product with potent antioxidant activity found in peanuts, berries and grapes [18]. Previous studies have reported that resveratrol has protective effects in various disease models, such as cardiovascular disease, diabetes, cancer and neurodegenerative diseases [18, 19]. In the erythroid precursor cells isolated from SCD patients, resveratrol increased the fetal hemoglobin (HbF) levels [20]. In animal models of erectile dysfunction, treatment with resveratrol restored endothelial function and reduced oxidative stress in CC [21–24].

**Fig 1:**
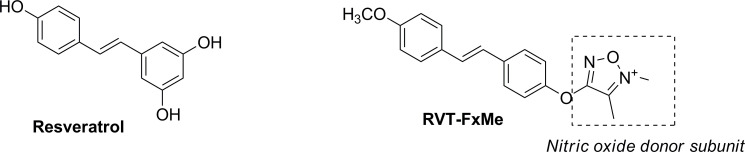
Chemical structures of resveratrol and RVT-FxMe.

Current pharmacological approaches are non-preventive for priapism [25]; therefore, developments of preventive strategies are necessaries. Preferably, pharmacological strategies should correct the pathophysiologic basis of this disorder. Since low NO-cGMP bioavailability and oxidative stress are associated to priapism, we have developed the hybrid compound (*E*)-4-(4-(4-methoxystyryl) phenoxy)-3-methyl-1,2,5-oxadiazole 2-*N*-oxide (RVT-FxMe), derived from resveratrol bearing a NO-donor subunit (Fig 1). RVT-FxMe given by oral route exhibited antinflammatory/analgesic effects *in vivo* and reduced up to 64.3% the levels of TNF-α in the supernatants of macrophages that were previously stimulated with LPS. Moreover, RVT-FxMe induced gamma-globin chains (γG + γA) in CD34+ cells, demonstrating a potential to induce HbF, an important therapeutic intervention for SCD. Differently from resveratrol, known by its ability to induce membrane perturbation, RVT-FxMe did not act by unspecific mechanisms. In addition, it was not mutagenic and genotoxic, representing a new prototype to treat SCD symptoms [26].

The aim of this study was to evaluate the effects of RVT-FxMe, on functional alterations of erectile function in murine models that display low NO-cGMP bioavailability and increased oxidative stress, SCD transgenic mice and eNOS^-/-^ mice. We have focused on the dysregulated NO-cGMP pathway in erectile tissue of SCD and eNOS^-/-^ mice.

## Materials and methods

### Ethics statement

All experimental procedures in this study were carried out in accordance with the general ethical guidelines for animal use established by the Brazilian Society of Laboratory Animal Science (SBCAL) and EC Directive 86/609/EEC for Animal Experiments and were approved by an institutional Committee for Ethics in Animal Experimentation of the University of Campinas (IACUC/CEEA-UNICAMP, Permit number 5729-1/2021). Mice were anesthetized with 100 mg/kg Ketamine + 10 mg/kg Xylazine by intraperitoneal injection and all efforts were made to minimize animal suffering.

### Animals and treatment

Three- to five-month-old wild type (WT, C57BL/6), Townes SCD transgenic and eNOS^-/-^ male mice were treated with compound RVT-FxMe (25 mg/Kg/day) or its vehicle (20% Cremophor®) daily for 2 weeks via intraperitoneal injections. The mice were obtained from Jackson Laboratories (Bar Harbor, ME) and were generated and characterized at the Multidisciplinary Center for the Investigation of Biological Science in Laboratory Animals of University of Campinas. The homozygous Townes transgenic sickle cell mouse is a “knock-in” model that was developed by the substitution of the mouse α-globin genes by human α-globin genes, while the mouse β-globin genes are substituted by human Aγ and βS (sickle) globin genes [5].

### Hematological parameters

Hematological parameters were performed on ethylenediamine tetra acetic acid-anticoagulated blood at 30 minutes after blood collection. Whole blood was collected by intracardiac puncture from ketamine/xylazine-anesthetized mice. Blood count was performed using a Sysmex XN-3000™ (Sysmex, Kobe, Japan).

### Plasma hemoglobin measurement

Mice plasma samples were used to quantify the hemoglobin through colorimetric assays using the Hemoglobin Colorimetric Assay (Cayman Chemical, Ann Arbor, Michigan), according to the manufacturer protocol. Assays were done in duplicate.

### Functional studies in cavernosal strips and concentration-response curves

Strips of CC obtained from mice anesthetized with ketamine and xylazine were mounted in a 5-mL organ system containing Krebs-Henseleit solution (mM: 117 NaCl, 4.7 KCl, 2.5 CaCl_2_, 1.2 MgSO_4_, 1.2 KH_2_PO_4_, 25 NaHCO_3_ and 11 glucose) at 37°C and continuously bubbled with a mixture of 95% O_2_ and 5% CO_2_ (pH 7.4). Changes in isometric force were recorded using a strip myograph for isometric force recording (Danish Myo Technology, Model 610M, Denmark) coupled with an acquisition system (PowerLab 8/30, LabChart 7, ADInstruments, Sydney-NSW, Australia). The resting tension was adjusted to 2.5 mN at the beginning of the experiments. The equilibration period was 60 min and the bathing medium was changed every 15 min until the start of the experiments. Cumulative concentration-response curves were constructed for both the muscarinic agonist acetylcholine (ACh, 10^−9^ to 10^−5^ M) and the NO-donor compound sodium nitroprusside (SNP; 10^−9^ to 3 x 10^−5^ M) in tissue strips pre-contracted with phenylephrine (3 × 10^−6^ to 10^−5^ M). EC_50_ values are presented as the negative logarithm (pEC_50_), and calculated by a fitting concentration–response relationship to a sigmoidal model of the form log-concentrations vs response using the GraphPad Software (GraphPad Software, San Diego, CA, USA).

### Electrical-field stimulation (EFS) in corpus cavernosum strips

EFS was applied to the cavernosal strips placed between two platinum electrodes connected to a Grass S88 stimulator (Astro-Med Industrial Park, RI, USA). EFS was conducted at 50 V, 1 ms pulse width and trains of stimuli lasting 10 sec at varying frequencies. Frequency-response relationships were investigated at supra maximum voltage in all preparations stimulated electrically. In order to study the nitrergic cavernosal relaxations, tissues were pretreated with guanethidine (3 × 10^−5^ M; to deplete the catecholamine stores of adrenergic fibers) and atropine (10^−6^ M; to produce muscarinic receptor antagonism) prior to pre-contraction with phenylephrine (3 × 10^−6^ to 10^−5^ M). When a stable contraction level was attained, a series of EFS-induced relaxations were constructed (1–32 Hz). Data were calculated relative to the maximal changes from the contraction produced by phenylephrine in each tissue, which was taken as 100%.

### Determination of cGMP levels

Quantitative assays for cGMP were performed using a commercial enzyme immunoassay kit (Cayman Chemical Cyclic GMP EIA kit, Ann Arbor, MI, USA). For penile cGMP content, frozen penile tissue was homogenized in 5% trichloroacetic acid and centrifuged. TCA was extracted from the supernatant with three washes of water-saturated ether. cGMP was expressed as pmol/mg tissue.

### Statistical analysis

The program GraphPad Prism (GraphPad Software Inc., San Diego, CA, USA) was used for statistical analysis. Data are expressed as the mean ± S.E.M. of N experiments. Statistical comparisons were made using one-way analysis of variance (ANOVA), and the Tukey method was chosen as a post-test. A value of *P* < 0.05 was considered statistically significant.

## Results

### Hematological parameters

**Fig 2:**
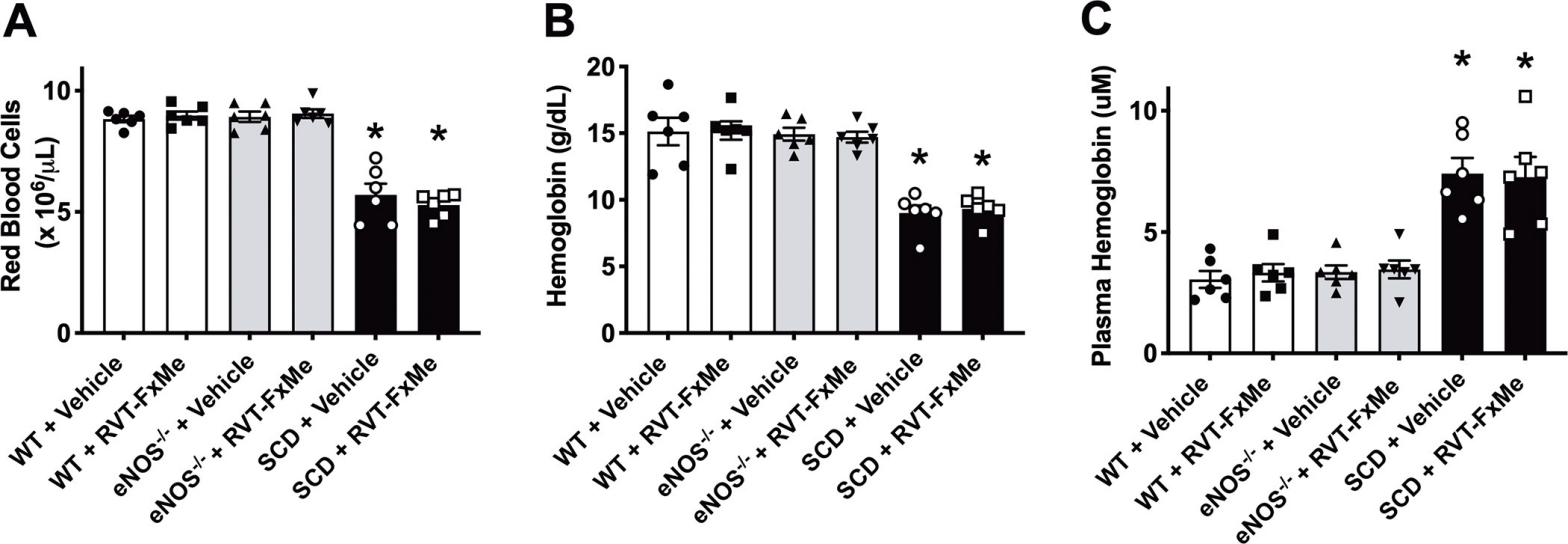
Hematological parameters of WT, eNOS^-/-^ and SCD mice treated with RVT-FxMe (25 mg/kg/day, 2 weeks) or vehicle. Data are shown as mean ± SEM of 6 mice per group. (A) Red blood cell, (B) hemoglobin and (C) plasma hemoglobin. One-way ANOVA: *P < .05 vs WT group.

Reduced red blood cells (Fig 2A) and total hemoglobin (Fig 2B) indicate that SCD have severe anemia compared to WT and eNOS^-/-^ mice. Plasma hemoglobin was significantly higher (*P* < .05) in SCD compared to WT and eNOS^-/-^ mice (Fig 2C). Compound RVT-FxMe treatment did not change red blood cell (Fig 2A), total hemoglobin (Fig 2B) and plasma hemoglobin (Fig 2C) in WT, SCD and eNOS^-/-^ mice.

### Compound RVT-FxMe treatment reversed the increased cavernosal relaxation in eNOS^-/-^ mice

**Fig 3:**
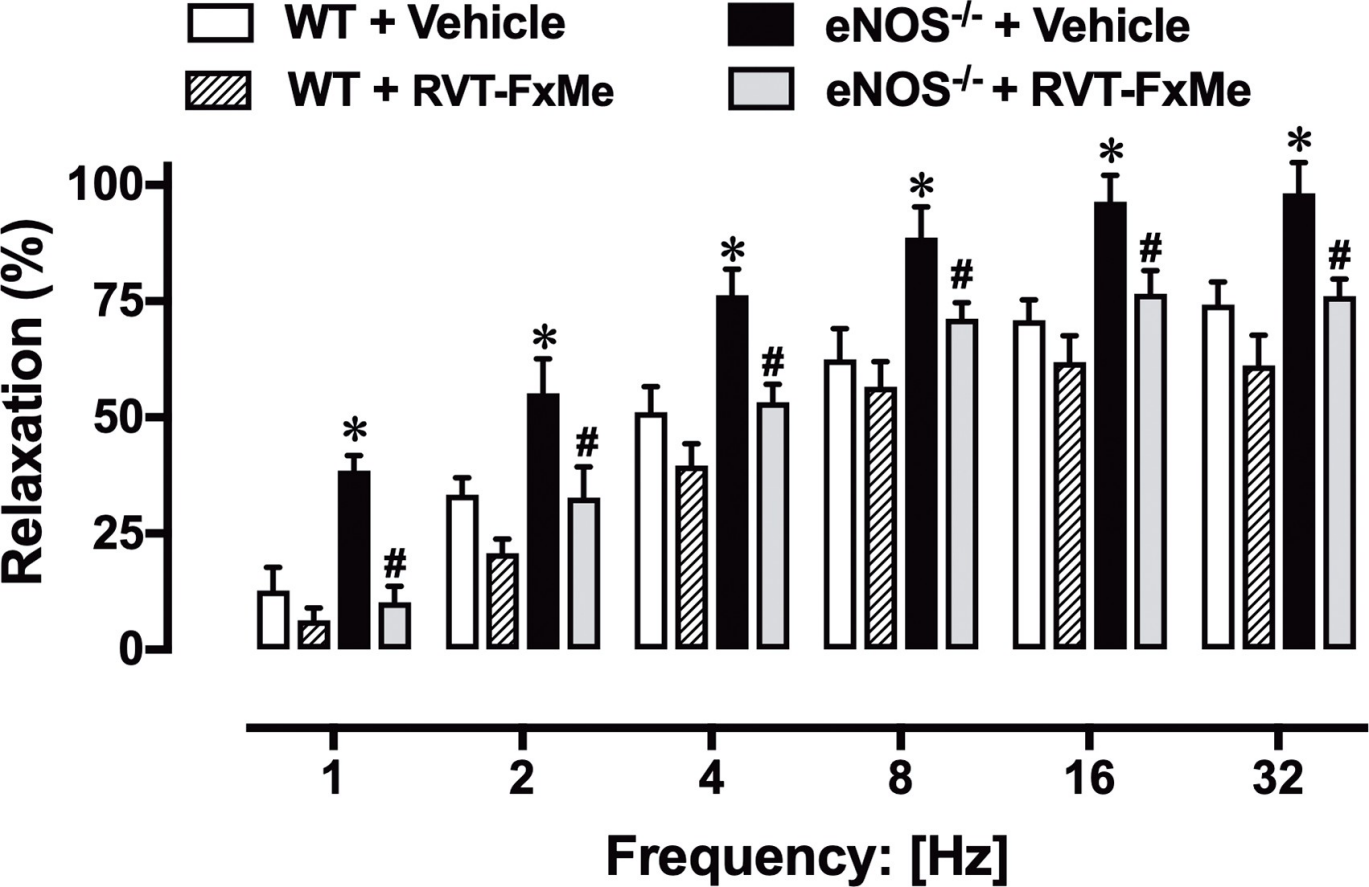
Relaxation responses to electrical-field stimulation (EFS) in corpus cavernosum strips from WT and eNOS^-/-^ mice treated with RVT-FxMe (25 mg/kg/day, 2 weeks) or vehicle. Data were calculated relative to the maximal changes from the contraction produced by phenylephrine (10^−5^ M) in each tissue, which was taken as 100%. Data represent the mean ± SEM for 6 mice in each group. One-way ANOVA: *P < .05 vs WT + Vehicle, # *P* < .05 vs respective vehicle group.

EFS induced frequency-dependent CC relaxations, which were significantly higher (*P* < .05) in eNOS^-/-^ compared to WT mice at all frequencies tested (Fig 3). Compound RVT-FxMe treatment normalized to WT values the nitrergic relaxations in eNOS^-/-^ mice (Fig 3). No significant changes after RVT-FxMe treatment were observed in EFS-induced CC relaxations of WT mice (Fig 3).

**Fig 4:**
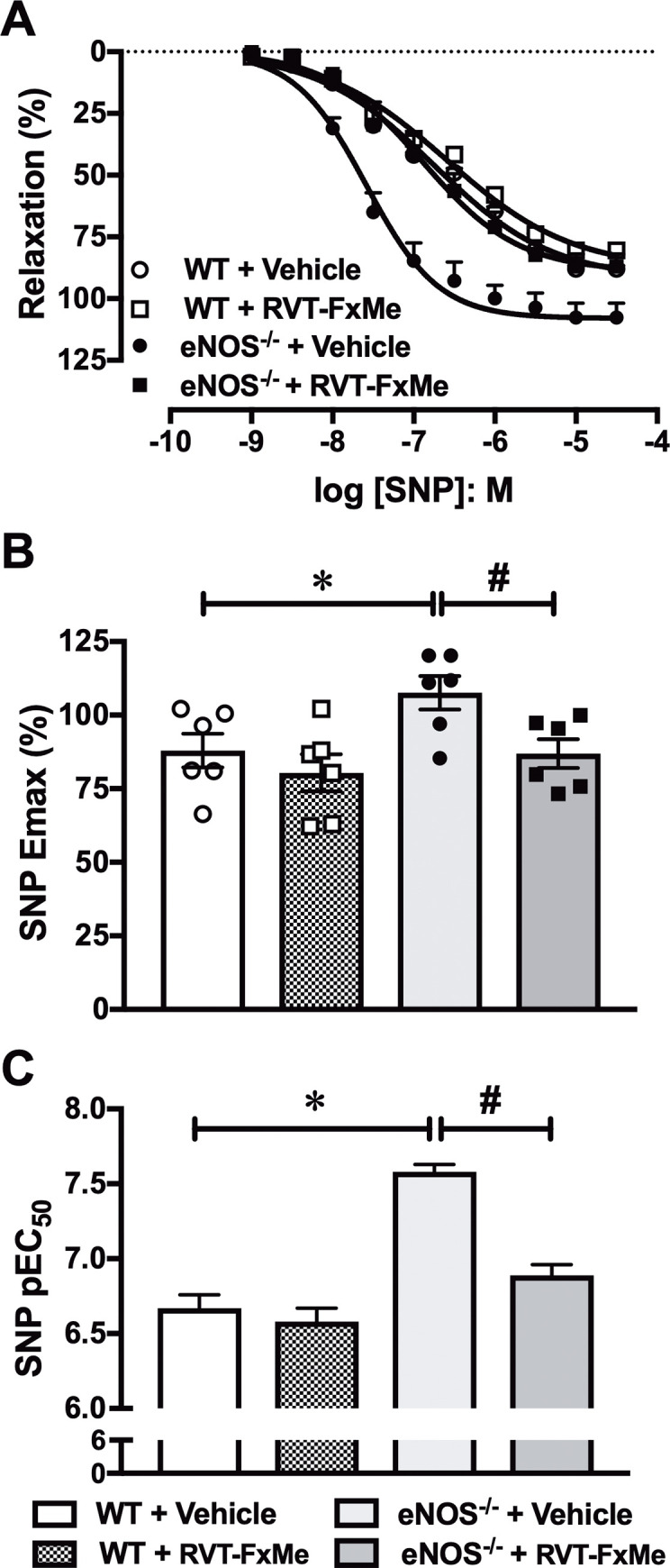
Concentration-response curves to sodium nitroprusside (SNP) in corpus cavernosum strips from WT and eNOS^-/-^ mice treated with RVT-FxMe (25 mg/kg/day, 2 weeks) or vehicle. Data were calculated relative to the maximal changes from the contraction produced by phenylephrine (10^−5^ M) in each tissue, which was taken as 100%. Data represent the mean ± SEM for 6 mice in each group. One-way ANOVA: *P < .05 vs WT + Vehicle, # *P* < .05 vs respective vehicle group.

The cumulative addition of SNP (1 nM—30 μM) produced concentration-dependent relaxations in CC from WT and eNOS^-/-^ groups (Fig 4A). SNP maximal response (Fig 4B) and pEC_50_ (Fig 4C) values were significantly higher (*P* < .05) in CC from eNOS^-/-^ compared to WT mice. Compound RVT-FxMe treatment also fully normalized to WT values the SNP maximal response and pEC_50_ in eNOS^-/-^ mice. No significant changes after RVT-FxMe treatment were observed in SNP-induced CC relaxations of WT mice (Fig 4A).

### Compound RVT-FxME treatment did not modify the increased cavernosal relaxation in SCD mice

**Fig 5:**
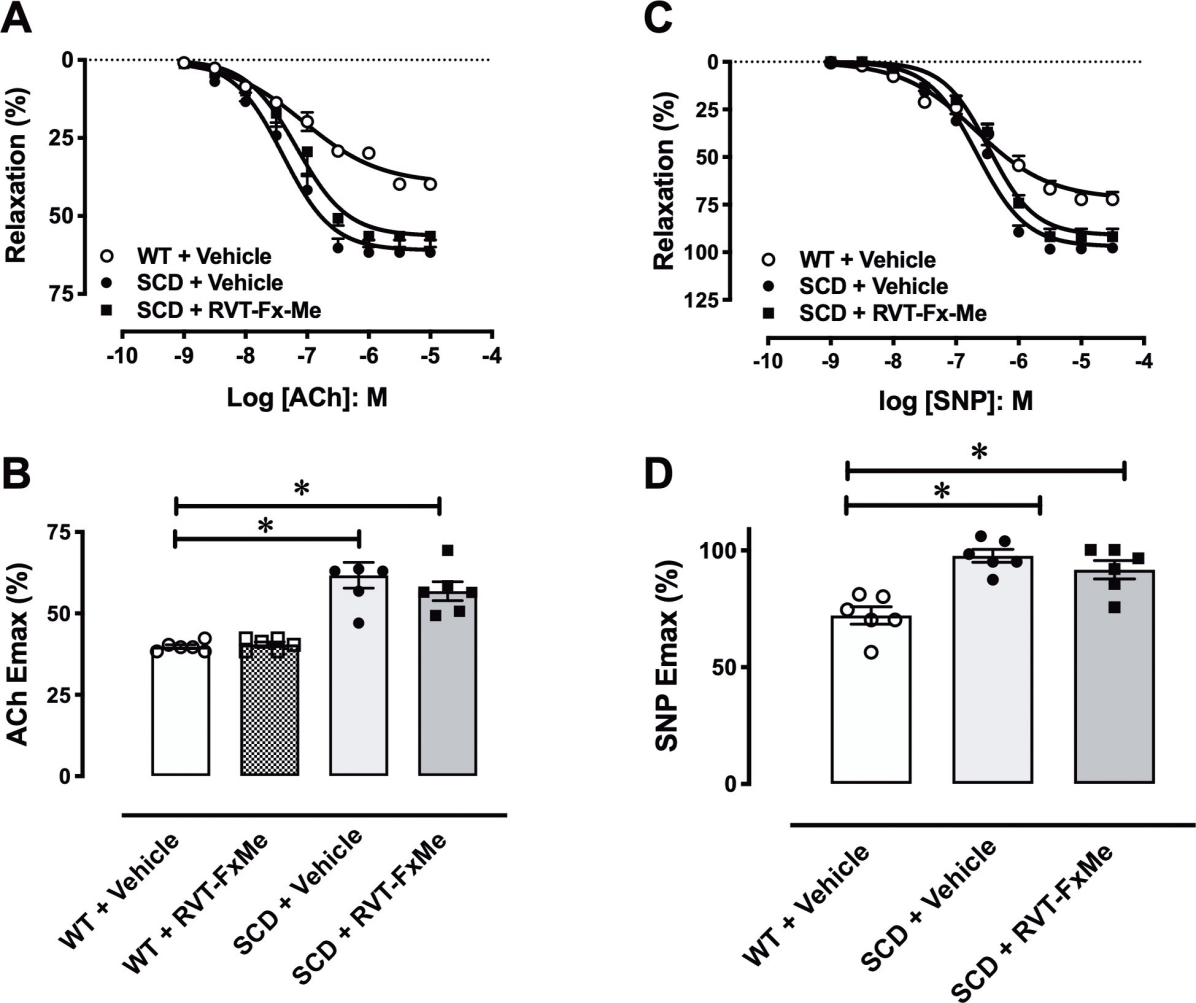
Concentration-response curves to acetylcholine (ACh) sodium nitroprusside (SNP) in corpus cavernosum strips from WT and SCD mice treated with RVT-FxMe (25 mg/kg/day, 2 weeks) or vehicle. Data were calculated relative to the maximal changes from the contraction produced by phenylephrine (10^−5^ M for WT mice and 3 × 10^−6^ M for SCD mice) in each tissue, which was taken as 100%. Data represent the mean ± SEM for 6 mice in each group. One-way ANOVA: *P < .05 vs WT + Vehicle.

The cumulative addition of ACh (Fig 5A) and SNP (Fig 5C) to PE-contracted tissues produced concentration-dependent relaxations in WT and SCD mice. ACh (Fig 5B) and SNP maximal response (Fig 5D) values were significantly higher (*P* < .05) in CC from SCD compared to WT mice. In SCD mice treated with RVT-FxME, ACh (Fig 5B) and SNP maximal response (Fig 5D) values were also significantly higher (*P* < .05) compared to WT mice, but without changing compared to SCD+Vehicle.

**Fig 6:**
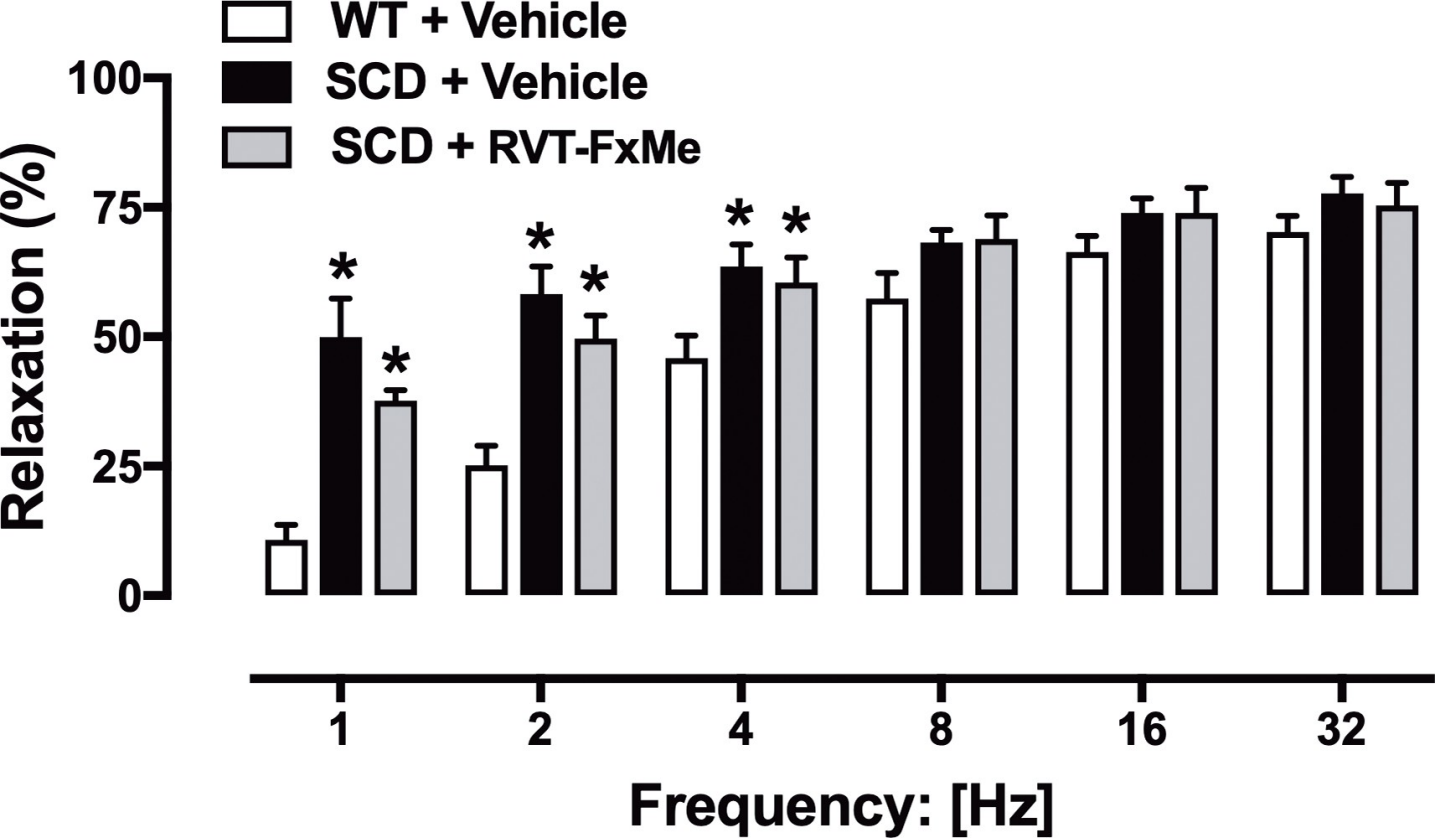
Relaxation responses to electrical-field stimulation (EFS) in corpus cavernosum strips from WT and SCD mice treated with RVT-FxMe (25 mg/kg/day, 2 weeks) or vehicle. Data were calculated relative to the maximal changes from the contraction produced by phenylephrine (10^−5^ M for WT mice and 3 × 10^−6^ M for SCD mice) in each tissue, which was taken as 100%. Data represent the mean ± SEM for 6 mice in each group. *P < .05 vs WT + Vehicle.

CC relaxations to EFS were significantly higher (*P* < .05) in SCD compared to WT mice, as observed at 1, 2 and 4 Hz (Fig 6). In SCD mice treated with RVT-FxME, CC relaxations to EFS were also significantly higher (*P* < .05) compared to WT mice (Fig 6).

### Effect of RVT-FxME on cGMP levels in the penises

**Fig 7:**
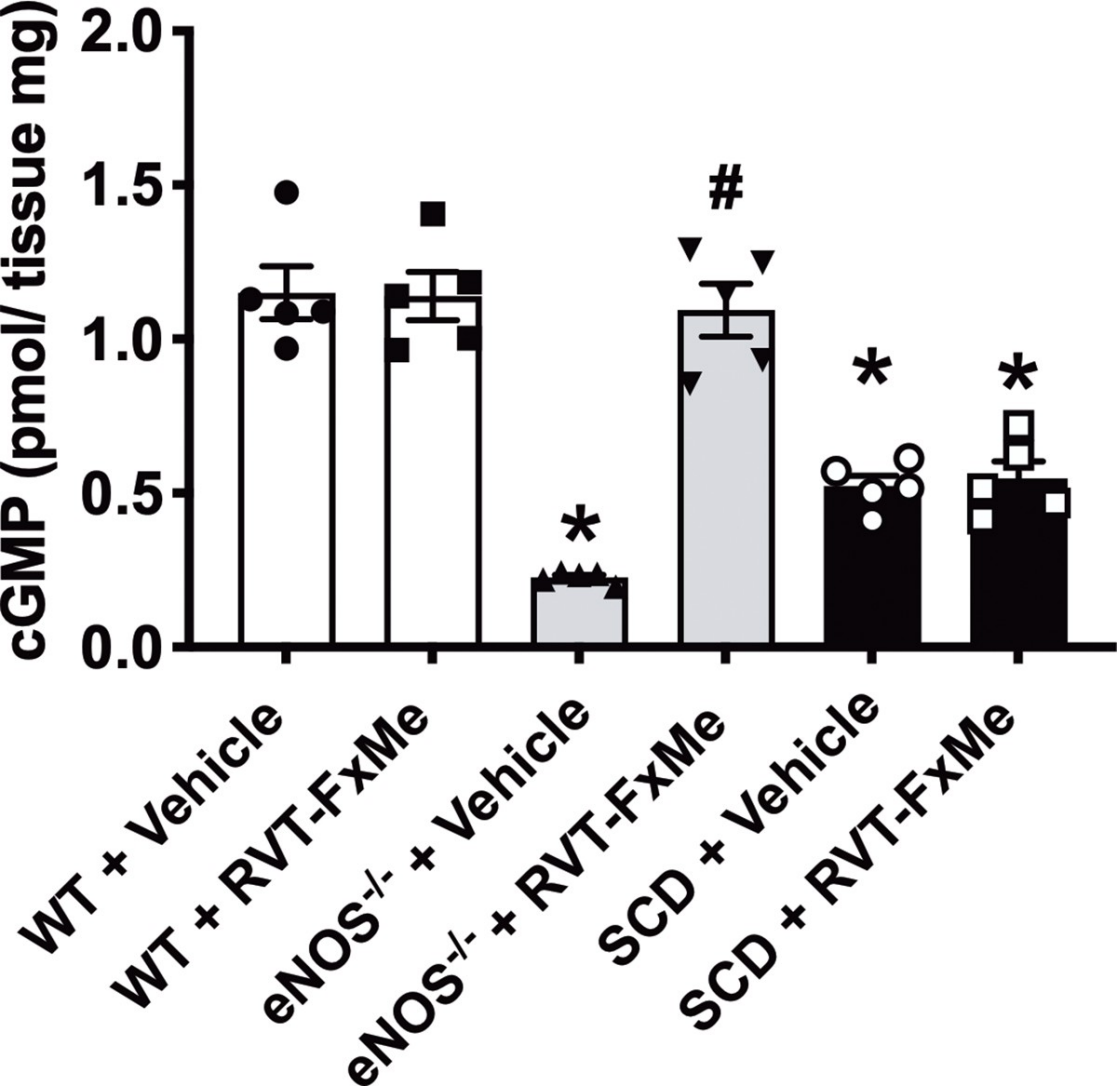
cGMP levels in penises of WT, eNOS^-/-^ and SCD mice treated with RVT-FxMe (25 mg/kg/day, 2 weeks) or vehicle. Data represent the mean ± SEM for 5 mice in each group. One-way ANOVA: *P < .05 vs WT + Vehicle, # *P* < .05 vs respective vehicle group.

The basal cGMP content in the erectile tissue was 81% and 53% lower (P < 0.05) in penises of eNOS^-/-^ and SCD mice compared with WT mice (Fig 7). Compound RVT-FxMe treatment also fully normalized to WT values the cGMP levels in eNOS^-/-^ mice, with no changes in the WT and SCD group (Fig 7).

## Discussion

PDE5 gene expression is regulated positively by cGMP in CC smooth muscle cells [27]. Lower NO-cGMP production in CC from eNOS^-/-^ and SCD mice leads to downregulation PDE5 and exaggerated CC relaxation, which is priapism phenotype [6, 12, 28]. In CC from SCD mice and patients, low NO production is related to decreased eNOS activity [7, 28, 29]. Oxidative stress is also amplified in the SCD penis due to upregulation of expression of NADPH oxidase subunit gp91phox and eNOS uncoupling [6, 11, 29]. Therefore, we evaluated the treatment with RVT-FxMe on functional alterations of erectile function in two murine models that display low NO-cGMP bioavailability and elevated oxidative stress, SCD and eNOS^-/-^ mice.

In our study, in accordance with previous studies [12], baseline cGMP levels were lower in the penises of the eNOS^-/-^ group. Treatment with RVT-FxMe normalized baseline cGMP levels in the eNOS^-/-^ group. These results indicate that treatment with RVT-FxMe was efficient in normalizing the bioavailability of NO in the penis of the eNOS^-/-^ mice group. It is likely that the NO released by the RVT-FxMe activates sGC in CC, resulting in the production of cGMP. A previous study reported that treatment with a compound that activates sGC normalized baseline cGMP levels in the penises of mice with low NO bioavailability [30].

We evaluated the CC relaxations induced by EFS and SNP. Stimulation of nitrergic fibers by EFS induces release of NO that diffuses to CC smooth muscle cells where it activates sGC [2]. SNP is a compound that releases NO and relaxes cavernosal smooth muscle by cGMP-dependent mechanisms. In our study, EFS- and SNP-induced cavernosal relaxations were significantly increased in eNOS^-/-^ mice group, which were restored to WT values by the treatment with RVT-FxMe. Previous study has shown that increased CC relaxant responses is due decreased PDE5 activity in the penises from SCD and eNOS^-/-^ mice [6, 7, 11, 12]. Therefore, it is presumably likely that RVT-FxMe treatment restored relaxant response in eNOS^-/-^ mice due to normalization of PDE5 activity in cavernosal tissue. A limitation of our study is that we did not measure PDE5 in penises of eNOS^-/-^ mice. RBCs, total hemoglobin and plasma hemoglobin were not changed by RVT-FxMe treatment in eNOS^-/-^ mice, indicating that the effects observed on the penises were not caused by changes in hematological parameters.

ACh activates the muscarinic receptor on the endothelial cell of CC, resulting in activation of eNOS, which converts L-arginine to NO. EFS promotes activation of nNOS in nitrergic fibers, which also converts L- arginine to NO. The NO generated by eNOS and nNOS diffuses to CC smooth muscle cells promoting its relaxation [2]. Previous study reported that penises SCD mice display lower expression/activity of eNOS, but no change in nNOS [7, 10, 29]. Thrombospondin-1 (TSP1), matricellular protein, inhibits eNOS and is elevated in patients and mice with SCD [31–33], but no studies have investigated the expression and function of TSP1 in the penis. SCD mice exhibited an excessive CC relaxant response induced by ACh, EFS and SNP (this study and [6, 7, 11]). Increased erectile tissue relaxation is associated with low PDE5 expression in the penis of SCD mice [7, 10, 11, 29]. Two-week treatment with RVT-FxMe did not change the increased relaxant responses to ACh, EFS and SNP in CC from SCD group. These functional results indicate that the treatment with RVT-FxMe did not modify the enzymes that are active by the action of ACh, EFS and SNP in the penis of the SCD group, such as eNOS, nNOS, sGC, PKG and PDE5.

In the SCD, the process of intravascular hemolysis leads to the release of free hemoglobin into the plasma as RBCs rupture [3]. In blood plasma, haptoglobin binds to free hemoglobin forming a complex, which is metabolized by macrophages in the reticuloendothelial system [34]. However, in SCD, high concentrations of hemoglobin are released into the plasma, saturating haptoglobin, and thus accumulating free hemoglobin in the plasma [35, 36]. Oxyhemoglobin (HbFe^2+^) in plasma or in the interstitial space reacts with NO, generating nitrate (NO^3+^) and methemoglobin (HbFe^3+^) [36, 37]. In our study, in accordance with previous studies [29], baseline cGMP levels were lower in the penises of the SCD group. Treatment with RVT-FxMe did not change cGMP levels in the SCD group. This result indicates that the increased concentration of oxyhemoglobin in the plasma is inactivating the NO donated by the compound RVT-FxMe before it reaches sGC in CC smooth muscle cells from SCD mice, thus impairing its pharmacological effect.

While mechanistic data with promising potential is showed, the current study is not without limitations. RVT-FxMe dose titration and effects in the mid- and long-term warrant complementary studies. Future studies are needed to evaluate the effects of treatment with RVT-FxMe on proteins from NO-sGC-cGMP-PDE5 pathway and levels of reactive oxygen species in the penises of SCD and eNOS^-/-^ mice.

## Conclusion

Compound RVT-FxMe short-term treatment reversed the enhanced NO-cGMP-mediated CC relaxations in eNOS^-/-^ mice, but not in SCD mice. It is likely that excess of plasma hemoglobin in SCD mice acts to inactivate NO before it reaches sGC, avoiding restoration of NO bioavailability in penis.
